# Three methods to monitor utilization of healthcare services by the poor

**DOI:** 10.1186/1475-9276-8-29

**Published:** 2009-08-04

**Authors:** Abbas Bhuiya, SMA Hanifi, Farhana Urni, Shehrin Shaila Mahmood

**Affiliations:** 1 Social and Behvioural Sciences Unit, Public Health Sciences Division, ICDDR, B, Mohakhali, Dhaka 1212, Bangladesh

## Abstract

**Background:**

Achieving equity by way of improving the condition of the economically poor or otherwise disadvantaged is among the core goals of contemporary development paradigm. This places importance on monitoring outcome indicators among the poor. National surveys allow disaggregation of outcomes by socioeconomic status at national level and do not have statistical adequacy to provide estimates for lower level administrative units. This limits the utility of these data for programme managers to know how well particular services are reaching the poor at the lowest level. Managers are thus left without a tool for monitoring results for the poor at lower levels. This paper demonstrates that with some extra efforts community and facility based data at the lower level can be used to monitor utilization of healthcare services by the poor.

**Methods:**

Data used in this paper came from two sources- Chakaria Health and Demographic Surveillance System (HDSS) of ICDDR,B and from a special study conducted during 2006 among patients attending the public and private health facilities in Chakaria, Bangladesh. The outcome variables included use of skilled attendants for delivery and use of facilities. Rate-ratio, rate-difference, concentration index, benefit incidence ratio, sequential sampling, and Lot Quality Assurance Sampling were used to assess how pro-poor is the use of skilled attendants for delivery and healthcare facilities.

**Findings:**

Poor are using skilled attendants for delivery far less than the better offs. Government health service facilities are used more than the private facilities by the poor.

Benefit incidence analysis and sequential sampling techniques could assess the situation realistically which can be used for monitoring utilization of services by poor. The visual display of the findings makes both these methods attractive. LQAS, on the other hand, requires small fixed sample and always enables decision making.

**Conclusion:**

With some extra efforts monitoring of the utilization of healthcare services by the poor at the facilities can be done reliably. If monitored, the findings can guide the programme and facility managers to act in a timely fashion to improve the effectiveness of the programme in reaching the poor.

## Background

Achievement of equity by way of improving the condition of the poor and disadvantaged in all aspects of life including health is one of the core goals of the contemporary development paradigm. It has been argued that unless performance indicators are examined by socioeconomic status of the population, improvement in average statistics may hide the presence of persistent or worsening inequities in a society [1]. This clearly indicates the need for monitoring the health and development indicators by socioeconomic status of the population. However, the challenge is to generate healthcare utilization data by socioeconomic status of the population with an acceptable level of statistical precision and reporting them regularly in an easily understandable fashion. National level healthcare utilization data often collected through cross sectional surveys, if analysed by the socioeconomic status of the population, can only portray the average level of disparities at the national level. Furthermore, this does not necessarily allow identification of inadequately performing regions, sub-regions, or lower level administrative or programme units with respect to reaching the poor. Thus, national level data serve a limited purpose for the facility/programme managers to assess the situation at the lowest level where most of the actions have to take place to improve the situation. Using routinely collected data from the facilities and communities through systems, such as, Health and Demographic Surveillance System (HDSS), utilization of healthcare services by the poor can be monitored at the local level. The 40 INDEPTH member sites in the developing world are uniquely placed to adopt the monitoring system to influence programmes and policies for enhancing utilization of health services by the poor [2]. Despite this potential, HDSS or similar other systems so far has put limited attention to monitoring utilization of health services by the poor at the local level. One of the reasons could be lack of attempts and demonstration of the methodological options available to do so. It is against this background that this paper is written.

## Methods and materials

### The Study Area

The paper is based on data collected from Chakaria, a remote rural area in the south-east coast of Bangladesh. Chakaria is one of the 508 sub-districts in the country with a population of around 420,000. The area is a typical of rural Bangladesh with agriculture as the main occupation of its inhabitants. The infant mortality rate in the area during 2007 was 48 per 1,000 live births. Life expectancy was 69.7 years for females and 67.2 years for males. Total fertility rate was 3.5 children per woman. 95% of the deliveries during 2007 took place at home and only 19% of all the deliveries were assisted by skilled attendants [3]. The sub-district headquarters has one 31 bed primary care government hospital, three private clinics and an NGO hospital with inpatient and outpatient services. Primary health care services are provided through 13 primary healthcare centres run by the government. In addition, private services of nearly 40 physicians and 300 informal healthcare providers practicing modern medicine are available outside the institutional services [4].

### Data Sources

Data collected from the households through quarterly visits as a part of Chakaria Health and Demographic Surveillance System (HDSS) during 2005–2007 and from government and private health facilities in Chakaria during March–June 2006 were used. Chakaria HDSS with its explicit focus on the poor and vulnerable, regularly collect data on ownership of household asset, occupation of main income earner and land owned by the household [3]. Chakaria is a member of INDEPTH network [2].

### Categorization of Poor

Household socioeconomic status was assessed by asset score based on assets owned by any member of the household. The list of assets included television, radio, clock/watch, bedstead, phone, quilt, bi-cycle, wardrobe, and table/chair. In computing the asset index assets were assigned weights using the Principal Component Analysis (PCA) [5-7]. Despite some of its limitations, in many instances PCA has been recommended over the other alternatives for assigning weights in constructing asset index [8]. Households were categorized into quintiles based on the asset score. Proportion of households in the asset quintiles varied between 18–22 percent in the community. For the sequential sampling and LQAS households belonging to the lowest two asset quintiles were referred to as poor. In all other cases households from the lowest quintile were defined as poor.

### Description of the Methods and their Operationalization

Two different approaches were used in monitoring utilization of skilled attendants for delivery and utilization of healthcare facilities for curative care by the poor. One was based on household level data and the other one on facility based data. Rates based methods used community based data and the Benefit Incidence, Sequential Sampling and LQAS used facility based data.

### Rate-Ratio, Rate Difference, Concentration Curve and Concentration Index

Proportions of women who utilized skilled assistance during delivery from households in the five asset quintiles were computed for the years 2005 to 2007. Ratios and differences were calculated between the proportion in the lowest and the highest asset quintiles. Concentration index and concentration curve was constructed based on proportions from all the five asset quintiles. The concentration curve and related concentration index provides a means of assessing the degree of inequality in the distribution of a health variable. The value of concentration index can vary between -1 to +1 and a concentration index having a value of zero would indicate complete health equality among the various socioeconomic groups. On the other hand, a negative value would indicate a concentration of the health variable among the poorest group and a positive value would indicate the opposite [9-11]. The concentration index expresses the inequality in health across the full spectrum of socioeconomic status. In contrast, the rate-ratio and the rate-difference between the poorest and the richest quintile does not take into account the health status of the three middle quintiles [12].

### Benefit Incidence Ratio

A one-week data, collected during March-May 2006, was plotted to analyse the Benefit Incidence Ratio. Asset scores were calculated for the patients by applying the same procedures as were done for the households. Information on ownership of assets similar to the one included in the HDSS was collected from the patients attending various facilities. The cut-off points for asset scores derived from the household level data were applied to the asset scores derived for the patients to categorize patients into quintiles. The proportions of patients in various quintiles were compared with 20% and any deviation from 20% would give an assessment of the extent to which the facilities have been serving the poor. This approach, commonly known as Benefit Incidence Ratio, has been in use for quite some time [13,14].

### Sequential Sampling

Sequential sampling is commonly used for quality control in the industrial sector. In sequential test procedures the sample size needed to make a decision is not known in advance but rather determined by the sample results. In the sequential method, sample information is processed and evaluated as it becomes available, rather than at the end of the sampling process, as is done in fixed sample methods. The procedure continues to collect information only until enough evidence is available to make a decision confidently. The procedure was first developed by Wald (1947) [15]. The procedure uses a likelihood ratio to determine, after each observation is made, whether enough information is available to accept or reject the null hypothesis. Let us assume that L_1 _represents the likelihood function of the sample result with *k *samples when the alternative hypothesis H_1 _is true, and let L_0 _represent the likelihood function when the null hypothesis H_0 _is true. The ratio L_1_/L_0 _is the likelihood ratio. Details of likelihood function, null hypothesis and alternate hypothesis can be found elsewhere [16,17], When this ratio is large, the evidence points to H_1_. When it is small, the evidence points to H_0_. Intermediate values are inconclusive. A sequential test can be performed by calculating L_1_/L_0 _after each new observation is available by applying the following (adopted from McWilliams [18]):

1. Stop with a reject H_0 _decision if L_1_/L_0 _> A (h_2_+sk);

2. Stop with an accept H_0 _decision if L_1_/L_0 _< B (-h_1_+sk); and

3. Continue to sample if B ≤ L_1_/L_0 _≤ A.

Boundary values of *A *and *B *are chosen to satisfy Type I and Type II error specifications for the hypothesis test. Letting *α *and *β *represent probabilities of these errors respectively, *A *and *B *can be calculated according to



The calculation of L_1_/L_0 _for each observation is tedious, but it can be shown mathematically that comparing L_1_/L_0 _to *A *and *B *for each observation is equivalent to comparing with *h*_2_+*sk *and -*h*_1_+*sk *respectively, where







In a plot of *dk *(cumulative number of non-conformities) versus *k *(observation) *dk *= -*h*_1_+*sk *and *dk *= *h*_2_+*sk *represent parallel lines, namely the "accept" and "reject" boundary lines. The test can be carried out by simply plotting *dk *versus *k *for each observation and continuing to sample until either the accept or the reject boundary is crossed and a decision is made. In practice, now-a-days, one can get the values calculated by using software and produce a table or a chart quite easily. Theoretically once the cumulative number of non-conformities falls in any one the two regions it can never change its direction and therefore it stays in that region irrespective of the number of additional non-conformities. For more details on sequential sampling one can consult Wald and McWilliams [15,18].

In our case the equivalent of non-conformities analogous to quality of industrial product was the number of patients from quintiles other than the lowest two quintiles. We performed the assessment at three levels of utilization by the non-poor: a) 20% as the lower limit and 40% as the upper limit (equivalent to 80% and 60% in terms of poor); b) 40% as the lower limit and 60% as the upper limit (equivalent to 60% and 40% in terms of poor); and c) 60% as the lower limit and 80% as the higher limit (equivalent to 40% and 20% in terms of poor). The calculation was done by using SISA software [19]. For instance, if we take the upper and lower boundaries of the poor patients based on 40% as lower threshold and 60% as upper threshold, it would mean if the proportion of poor attendees is more than 60% of the patients then the facility would be considered as serving the poor adequately. On the other hand, if the proportion of poor is less than 40% then the facility would be considered as inadequately serving the poor. If the proportion lies in between 40% to 60% then no decision about the adequacy/inadequacy of the facility in serving the poor could be made. This paper presents only the findings for the upper and lower thresholds for poor attendees at 60% and 40% respectively.

Data on ownership of assets included in the household survey were collected from the first and subsequent 99 patients attending the outdoor services in the sub-district public hospital and a private clinic. Data collection was stopped after interviewing 100 patients on a single day. Asset scores were calculated by applying the same procedure used in calculating asset scores for the households. The cut-off points for quintiles derived from the household survey were used in classifying the patients attending the facilities as poor. The cumulative number of non-poor patients attending the facility in a particular day was plotted against cumulative number of patients interviewed. The procedure stopped as soon as any of the boundary lines defining the rejection and acceptance regions, based on upper and lower thresholds for poor attendees at 60% and 40% respectively, was crossed.

### Lot Quality Assurance Sampling (LQAS)

Lot quality assurance sampling (LQAS) originated in the manufacturing factory for quality control purposes to help the manufacturers in determining whether a batch or lot of goods can be accepted or rejected under pre-determined specifications [20]. In LQAS, a defective article is defined as one that fails to conform to the specifications of one or more quality characteristics. A common procedure in LQAS is to consider each submitted lot of product separately and to base the decision of acceptance or rejection of the lot on the evidence of one or more samples chosen at random from the lot [21].

Any systematic plan for single sampling requires that three numbers be specified. One is the number of articles '*N*' in the lot from which the sample is to be drawn. The second is the number of articles '*n*' in the random sample drawn from the lot. The third is the acceptance number '*d*'. The acceptance number is the maximum allowable number of defective articles in the sample. More than '*d*' defectives will cause the rejection of the lot. For instance, if we have a situation with *N *= 50, *n *= 5, and *d *= 0, it implies that "Take a random sample of size 5 from a lot of 50. If the sample contains more than 0 defectives, reject the lot; otherwise accept the lot." LQAS uses binomial probability to calculate the probability of accepting or rejecting a lot.

To apply the above in the context of monitoring utilization of health services by the poor, let us assume that the proportion of poor among the patients attending the facility is *p*. In a health facility with an infinitely large number of users, the probability *P(a) *of selecting a number *a *of poor in a sample size *n *is calculated as:



Where *p *= the proportion of poor attending the health facility

*q *= (*1-p*)

*n *= the sample size

*a *= the number of individuals in the sample who are poor

*n-a *= the number of non-poor in the sample, usually denoted by *d*.

LQAS aids in choosing the sample size and the permissible value of *n-a *and interpreting the results. In order to use LQAS in the context of monitoring the utilization of a facility by the poor, the following five initial decisions must be made [22-24].

1. Firstly, the services to assess. This is selected by the health systems manager. In our case, let it be the attendance in the outdoor services.

2. Second, the facility to monitor (e.g., Upazila Health Complex (UHC), Union Health and Family Welfare Centre and the like).

3. Third, the target attendance to receive the services (e.g. any patient attending the facility, infants etc.).

4. Fourth, a triage system must be defined for classifying the level of usage by the poor as adequate, somewhat inadequate, and very inadequate. This needs to be decided by the programme managers, policy makers or other stakeholders related to the health service delivery.

5. Fifth, the levels of the provider and consumer risks (Provider risk is the probability of wrongly classifying a facility as very unsatisfactory which can put the reputation of the facility at risk; Consumer risk is the probability of wrongly classifying a very inadequately performing health facility as adequate which can put the poor in the area at health risk). In most cases it may be around 10–15%.

Using the information on the above five points, a series of operating characteristics (OC) curve (An OC curve depicts the probabilities of accepting a lot based on the proportion of non-conformance in the lot, the sample size, and the value of *d*, allowable non-conformances. An OC curve enables decision makers to examine the possible risks involved), or their corresponding probability tables can be constructed with the above binomial formula. From the OC curves, one can select the sample size (i.e. *n*) and the number of non-poor allowed (i.e. *d*) in the LQAS sample for a given level of provider and consumer risk before deciding that a health area has inadequate utilization by the poor.

Let us assume that a consensus has been reached among the various stakeholders of health service delivery in Bangladesh that facilities with 80% or more poor in their users can be considered as performing adequately. While facilities with 50% or less poor patients ought to be considered as very inadequately performing and be identified for attention. The ones in the mid-range 50% to 80% may be considered somewhat fine and for the time being they need no special attention. By using these information, probabilities of detecting "adequately performing" or "inadequately performing" health facilities can be calculated. Table 1 presents such probabilities along with provider and consumer risks for various combinations of sample sizes and maximum allowable non-poor patients in the sample.

**Table 1:** Example of application of the LQAS methodology

Sample size	No. in the sample non-poor	Probability of detecting health facilities with 80% poor as adequate	Probability of detecting health facilities with 50% poor as inadequate	Provider Risk	Consumer Risk	Total classification error
(n)	(d)	(a)	(b)	(1-a)	(1-b)	(1-a)+(1-b)
8	0	0.17	1	0.83	0	0.83

	1	0.50	0.96	0.50	0.04	0.54

	2	0.79	0.83	0.21	0.17	0.38*

	3	0.94	0.64	0.06	0.36	0.42

12	0	0.07	1.00	0.93	0.00	0.93

	1	0.28	1.00	0.73	0.00	0.73

	2	0.56	0.98	0.46	0.02	0.48

	3	0.80	0.93	0.21	0.07	0.28

	4	0.93	0.81	0.07	0.19	0.27*

	5	0.98	0.61	0.02	0.39	0.41

14	0	0.04	1	0.96	0	0.96

	1	0.20	1	0.80	0	0.80

	2	0.45	0.99	0.55	0.01	0.56

	3	0.70	0.97	0.30	0.03	0.33

	4	0.87	0.91	0.13	0.09	0.22*

	5	0.96	0.79	0.04	0.21	0.25

19	0	0.01	1	0.99	0	0.99

	1	0.08	1	0.92	0	0.92

	2	0.24	1	0.76	0	0.76

	3	0.46	1	0.54	0	0.55

	4	0.67	0.99	0.33	0.01	0.34

	5	0.84	0.97	0.17	0.03	0.20

	6	0.93	0.92	0.07	0.08	0.15*

	7	0.98	0.82	0.02	0.18	0.20

**28**	5	0.50	1	0.50	0	0.50

	6	0.68	1	0.32	0	0.32

	7	0.81	0.99	0.19	0.01	0.20

	8	0.91	0.98	0.09	0.02	0.11

	**9**	**0.96**	**0.96**	**0.04**	**0.04**	0.08*

	10	0.99	0.90	0.01	0.10	0.11

* - Optimal decision rule for a sample size.

Source: Adopted from Valadez 1991, p:73.

Probabilities in Table 1 were calculated using the binomial formula. In each case, the upper and lower thresholds of the triage system were 80% and 50% respectively. The values in Table 1 (the row in bold) imply that in a sample of 28, if there are 9 or more non-poor, then the facility can be classified as inadequately performing in terms of serving the poor under the assumed triage of proportions (50%–80%) of poor. Details of LQAS method and its applicability in monitoring programme performance can be found elsewhere [24,25].

In our case, LQAS was applied in three scenarios with three levels of proportions of the poor in the facilities. In the first scenario, if the proportion of attendees in the facilities from the lowest two quintiles is less than 20%, then the facility is considered inadequate. If the proportion is more than 40%, then the facility is considered to be adequately performing. If the proportion is between 20%–40%, then no decision can be made. Under the above scenario, a facility can be considered as inadequately performing if in a sample of 50 attendees there are 35 or more are from quintiles other than the lowest two quintiles. The magnitude of misclassification in this case would be 11%.

In the second scenario, if the proportion of attendees from the lowest two quintiles is less than 40% then the facility is to be considered as inadequately performing in serving the poor. If the proportion is more than 60% then the facility is to be considered as adequately serving the poor. If the proportion is in between 40%–60% then no clear decision can be made. Under this scenario a facility can be considered as inadequately performing if in a sample of 50 patients, 25 or more are from quintiles other than the lowest two quintiles. The magnitude of misclassification in this case would be 16%.

The third scenario was with 60% as the lower and 80% as the higher thresholds. Under this scenario, a facility can be considered as inadequately serving the poor if in a sample of 50 there are 14 or more patients from other than the lowest two quintiles. The magnitude of misclassification in this case would be 11%. Although LQAS was applied in all these three scenarios, results based on 40%–60% thresholds are presented here. Decision regarding the pro-poor nature of the facilities could be made on a daily basis.

## Findings

### Rate-Ratio, Rate-Difference, Concentration Curve, and Concentration Index

**Figure 1:**
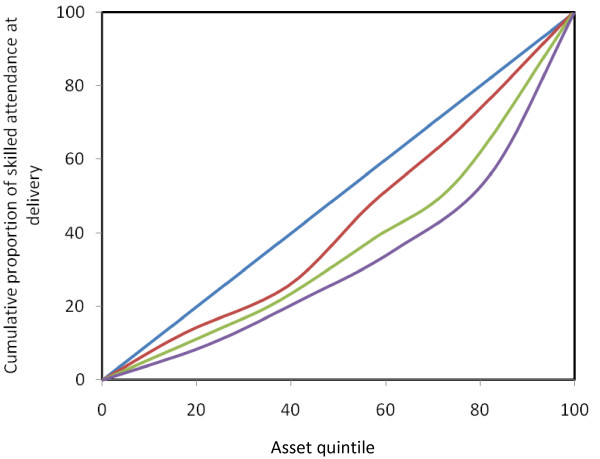
**Concentration Curves of inequality in the use of skilled assistance during delivery, Chakaria 2005–2007**. (Black line) – Line of equality (Blue line) – 2007 (Red line) – 2006 (Green line) – 2005

Table 2 presents the use of skilled birth attendants for delivery in Chakaria during 2005–2007 by asset quintiles. It can be seen that the use of skilled assistance during delivery has increased overtime among women from households in all the quintiles except in the highest quintile. The absolute difference in the use of skilled attendant between highest and lowest quintile has reduced from 24 percent in 2005 to 11 in 2007. In relative term the ratio of percent of utilization among the women from the highest quintile and the women from the lowest quintile has reduced from six in 2005 to two in 2007. A similar picture of reducing inequities is seen when one compares the value of concentration index over time. Figure 1 visually depicts the reduction in inequities as reflected by the reduction in the areas between the concentration curve and the line of equality.

**Table 2:** Percentage of women using skilled assistance during delivery, Chakaria 2005–2007

Asset quintile	2005	2006	2007
L(owest)	5.1	8.6	14.4

2	7.4	9.3	11.2

3	8.2	12.9	24.5

4	12.0	13.9	21.4

H(ighest)	29.6	28.6	25.4

Difference H-L	24.5	22.0	11.0

Ratio	5.8	3.3	1.8

Concentration Index	0.34	0.26	0.14

Figure 1 presents the concentration curves depicting the extent of inequalities in the use of skilled delivery assistance in Chakaria during 2005–2007. It can clearly be seen that the curves of inequality have been approaching the line of equality meaning a reduction in the level of inequalities over time.

### Benefit Incidence Ratio

**Figure 2:**
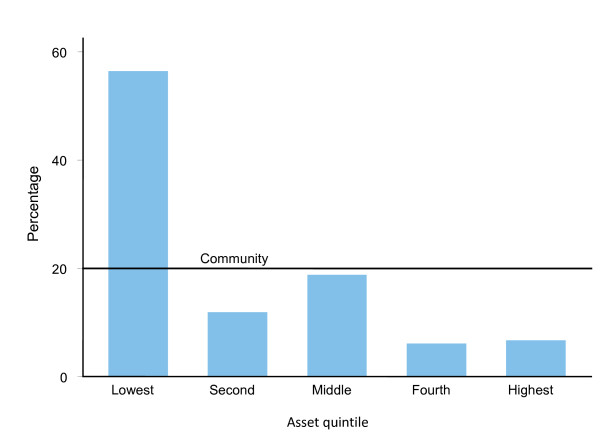
**Application of Benefit Incidence Ratio to assess whether a Govt. health facility is used adequately by the poor, Chakaria, March-May 2006**.

**Figure 3:**
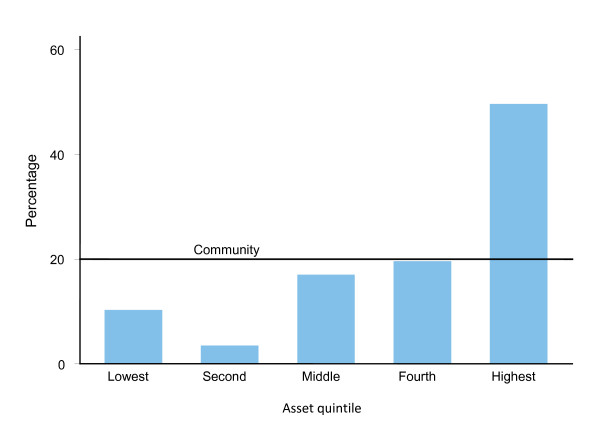
**Application of Benefit Incidence Ratio to assess whether a private health facility is used adequately by the poor, Chakaria, March-May 2006**.

Figure 2 and Figure 3 present distribution of patients attending a government facility and a private clinic respectively for outpatient services in Chakaria. The line termed "community" parallel to the horizontal axis represents an equal distribution of services among the various quintiles in the community. Any deviation from this line would indicate an unequal distribution of the services. Figure 2 shows that the patients in government facility were represented more by people from the lowest quintile than they were in the community. The situation in the private clinic was opposite of what was seen in the government facility (Figure 3).

### Sequential Sampling

**Figure 4:**
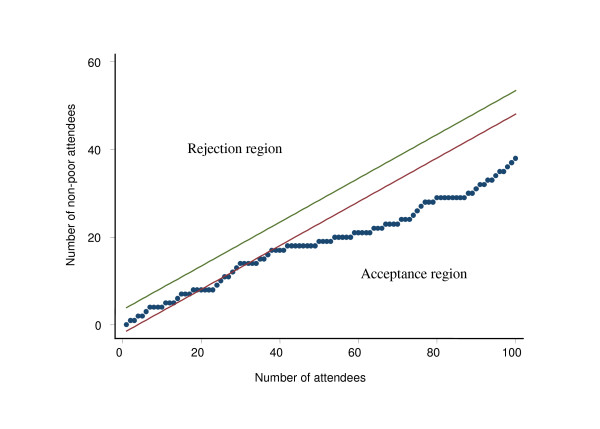
**Application of Sequential Sampling Scheme to decide whether a Govt. health facility is used by the poor adequately, Chakaria, June 2006**. Note: Results based on thresholds 40%–60%; alpha 5%; power 80%

**Figure 5:**
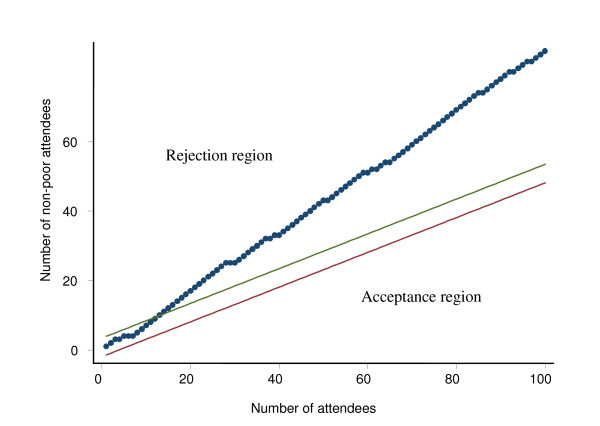
**Application of Sequential Sampling Scheme to decide whether a private health facility is used by the poor adequately, Chakaria, June 2006**. Note: Based on thresholds 40%–60%; alpha 5%; power 80%

Figure 4 and Figure 5 present results of the sequential sampling scheme for a government and a private facility in Chakaria respectively for the year 2006. It shows that the government facility was adequate in serving the poor with 40% and 60% as lower and upper thresholds of proportion of patients as poor and with 95% confidence level. These decisions for the government facility could be arrived at after interviewing the 42^nd ^patient. While for the private facility it required interviewing only 10 patients to conclude that the facility was inadequate on that week as it had less than 40% of the patients from the lowest two quintiles.

### Lot Quality Assurance Sampling

Table 3 presents number of non-poor patients found among the randomly chosen patients interviewed in the government and private facility. The application of LQAS in the Upazila Health Complex and the government facility resulted in terming the facility as serving the poor adequately or being pro-poor on all the six days of the interview week. The private clinic on the other hand failed in all the six days to be pro-poor. The exercise was based on 50 randomly chosen outdoor patients everyday for six days in a week. Of the 50 patients the maximum number of non-poor patients allowed was 25 with 40% and 60% lower and upper thresholds and 16% error of misclassification.

**Table 3:** Application of LQAS to assess whether health facilities are used by the poor adequately, Chakaria, June 2006

Period of evaluation Day	Threshold 40%–60%; Error = 16% Maximum number of non-poor (failure) permitted = 25			
	
	Upazila Health Complex		Private clinic	
	
	No. of non-Poor	Judgment	No. of non-Poor	Judgment
1	17	Pro-poor	42	Not pro-poor

2	13	Pro-poor	42	Not pro-poor

3	14	Pro-poor	45	Not pro-poor

4	13	Pro-poor	44	Not pro-poor

5	13	Pro-poor	43	Not pro-poor

6	21	Pro-poor	41	Not pro-poor

Note: based on information from 50 randomly chosen attendees

## Discussion

The analysis of the utilization of skilled delivery attendants by women from various socioeconomic statuses as measured by asset scores was the most straightforward and familiar one to the demographers and epidemiologists. The extent of inequity could be summarized by rate-ratio and/or rate-difference. One of the caveats in this approach is that neither the rate-ratio nor the rate-difference makes use of the information for all the asset quintiles- they only make use of the utilization rates of the women from the lowest and highest quintiles. One of the ways to tackle this problem would be to use concentration index (CI), which is another way of measuring the degree of inequalities. CI makes use of the information from all the asset quintiles. The other limitation of these approaches is their inability to infer about the fixed health facilities. Although facility specific information can be collected during HDSS rounds or cross sectional surveys, quite often with many sources of health care services an analysis of this kind will demand a large sample size to make inference about a facility. Moreover frequency of such assessment has to be limited to the frequency of the rounds and the collection and analysis of the data may not be done by the facility managers.

The benefit incidence, sequential sampling and LQAS methods can be applied at the facility level to provide information about the pro-poor nature of the services and compared to the community based approaches these methods can be applied more frequently as desired by the facility managers or the researchers with far less effort. Moreover the sample size required for these three methods are relatively smaller compared to community based surveys [20,26]. Nevertheless, there are issues associated with each of these methods which are discussed below.

The benefit incidence analysis showed the over representation of the attendees from the lowest two quintiles at the government facilities and under representation at the private facilities. However, it did not resort to formal statistical hypothesis testing in terms of identifying how big a deviation from 20% should be of concern. One can, of course, compare the proportions in the facility with 20% by using statistical tests. Such tests, however, would require the denominators from which the community proportion and the facility proportion were derived. In addition, a computation of the test statistics and associated probability to make an inference about the difference between the proportions of patients from the lowest quintiles would also be warranted. In case of LQAS and sequential sampling plans, the issue regarding how big a difference would be of significance is embedded in the procedure. In effect, the procedures operationalized those formal statistical testing in terms of number of non-conforming attendees, which in these cases, were from the quintiles other than the lowest two quintiles, with predetermined levels of error and power. The sequential plan has the advantage of plotting the cumulative number of non-poor against the number of attendees assessed for their socioeconomic status (SES) as they come, and provide a powerful visual tool for the facility managers. To have an equivalent visual representation in LQAS may not be that straightforward. Benefit incidence analysis, however, has the advantage of visual presentation without the formal statistical inference procedures built in. The caveat in the sequential sampling plan is that in some instances it may lead to a large number of interviews before a facility can be validly classified as pro-poor. This issue has been addressed in LQAS. LQAS combines the sequential test procedures with a fixed sample scheme in the sense that it allows decision-making by testing a fixed number of cases with a predetermined level of error.

In a situation leading to a non-stop examination of cases under sequential sampling, one can also resort to double sampling, meaning that if sequential sampling does not enable a decision making after examining a sample of cases then one can take another sample. However, more than two samples do not provide any additional advantage. LQAS has taken care of this issue of not being able to make a decision, for it combines sequential sampling and fixed sample methods. In case of LQAS, as we have seen, the number of attendees to be included in the sample is predetermined given the level of errors and thresholds, and thus it avoids the situation of no decision making. Methodologically speaking, the sequential plan and LQAS are almost similar with the above weaknesses and strengths. Either of them would serve the purpose of drawing inferences about the pro-poor nature of the services in terms of utilization by the poor. Facility management staff members can easily be trained to adopt any of these two methods.

Another challenge is the identification of the poor. We used asset quintiles for it allows the classification of attendees in terms of any interval such as deciles or quintiles, and in particular, allows the identification of the bottom twenty percent of the population. The challenge is to train facility managers to identify attendees from the lowest quintiles. This requires values for weights of assets and cut off points of asset scores based on the distribution of households in the community. Thus a community survey or an approximation from other surveys is required. Once the cut-off points are known, then the facility managers have to be trained in how to use the weights in calculating asset scores for the attendees, and how to use the cut-off points to identify attendees from the lowest quintiles. Easier alternatives exist that are simpler than using asset scores. These include using the number of assets owned, or other indicators such as land, occupation of main income earner, level of education and the like. The challenge in using these is to get deciles and quintiles. Use of indicators other than asset scores would obviously make the adoption of the monitoring system very attractive.

Another practical issue one has to deal with in adopting these methods is to decide how frequently the assessment should be made or, in other words, how frequently the data at the facility and the community level should be collected. The answer to the frequency of data collection at the community level is somewhat dependent on the chances of changes in the SES of the community. In many instances, the changes in SES are slow. The frequency of assessment at the facility level is dependent on the facility managers to some extent and also on the nature of services to be assessed. Again, it will largely depend on the nature of changes in the services or in the system. If the system is stable in terms of design, then perhaps, it is not useful to have very frequent assessments. If there is a special service for a short period and it is very important to make the service responsive to every section of the society, then perhaps it would be useful to increase the frequency of monitoring. The other issue to consider in deciding frequency of monitoring is the presence of a pattern during certain days, weeks, or months of the year when the facility is used by certain segments of the society more than usual. If such is the case, then this information should be used in deciding the timing and frequency of assessments. It may be mentioned that in the two upazilas where we worked, we examined the variation in use of the facilities by the SES of the attendees, and in most cases, no significant statistical variation was observed. This means that any day of the month would represent the pattern of the whole month satisfactorily.

## Conclusion

Benefit incidence analysis can be a starting point for monitoring the utilization of health services by the poor. Sequential sampling scheme allows a more formal inference about the performance in terms of utilization of services by the poor and its visual display of the findings on a continual basis makes the procedure quite attractive. LQAS may be preferable to sequential sampling for its ability to make a decision within a fixed sample size. Finally, HDSS sites or other data collection systems with data on socioeconomic status can examine health care utilization by the poor or any other marginalized group to assess how well health care services reach the poor and disadvantaged. This can guide the facility and programme managers to take appropriate actions in a timely fashion to ensure programmes reach the targeted population.

## Abbreviations

HDSS: Health and Demographic Surveillance System; UHC: Upazila Health Complex; LQAS: Lot Quality Assurance Sampling; OC: Operating Characteristics; CI: Concentration Index; SES: Socioeconomic Status.

## Competing interests

The authors declare that they have no competing interests.

## Authors' contributions

AB conceived the study, designed and implemented it. He also supervised data collection, analysis and drafted the manuscript. SMAH contributed in study design, supervised data collection and conducted the data analysis. He also contributed in drafting the manuscript. FU contributed in data analysis. SSM contributed in literature review for the manuscript and revising the manuscript. All authors read and approved the final manuscript.
